# Score performance of SAPS 2 and SAPS 3 in combination with biomarkers IL-6, PCT or CRP

**DOI:** 10.1371/journal.pone.0238587

**Published:** 2020-09-03

**Authors:** Michael Jahn, Jan Rekowski, Rolf Alexander Jánosi, Andreas Kribben, Ali Canbay, Antonios Katsounas

**Affiliations:** 1 Department of Nephrology, University of Duisburg-Essen, University Hospital Essen, Essen, Germany; 2 Institute for Medical Informatics, Biometry and Epidemiology, University of Duisburg-Essen, University Hospital Essen, Essen, Germany; 3 Department of Cardiology and Vascular Medicine, West German Heart and Vascular Center, University of Duisburg-Essen, University Hospital Essen, Essen, Germany; 4 Department of Medicine, Ruhr-University Bochum, Universitätsklinikum Knappschaftskrankenhaus Bochum GmbH, Bochum, Germany; University of Sao Paulo Medical School, BRAZIL

## Abstract

**Objective:**

We aimed to evaluate the effects of combining the Simplified-Acute-Physiology-Score (SAPS) 2 or the SAPS 3 with Interleukin-6 (IL-6) or Procalcitonin (PCT) or C-Reactive Protein (CRP) concentrations for predicting in-hospital mortality.

**Material and methods:**

This retrospective study was conducted in an interdisciplinary 22-bed intensive care unit (ICU) at a German university hospital. Within an 18-month period, SAPS 2 and SAPS 3 were calculated for 514 critically ill patients that were admitted to the internal medicine department. To evaluate discrimination performance, the area under the receiver operating characteristic curves (AUROCs) and the 95% confidence intervals (95% CIs) were calculated for each score, exclusively or in combination with IL-6 or PCT or CRP. DeLong test was used to compare different AUROCs.

**Results:**

The SAPS 2 exhibited a better discrimination performance than SAPS 3 with AUROCs of 0.81 (95% CI, 0.76–0.86) and 0.72 (95% CI, 0.66–0.78), respectively. Overall, combination of the SAPS 2 with IL-6 showed the best discrimination performance (AUROC 0.82; 95% CI, 0.77–0.87), albeit not significantly different from SAPS2. IL-6 performed better than PCT and CRP with AUROCs of 0.75 (95% CI, 0.69–0.81), 0.72 (95% CI, 0.66–0.77) and 0.65 (95% CI, 0.59–0.72), respectively. Performance of the SAPS 3 improved significantly when combined with IL-6 (AUROC 0.76; 95% CI, 0.69–0.81) or PCT (AUROC 0.73; 95% CI, 0.67–0.78).

**Conclusions:**

Our analysis provided evidence that the risk stratification performance of the SAPS 3 and, to a lesser degree, also of the SAPS 2 can increase when combined with IL-6. A more accurate detection of aberrant or dysregulated systemic immunological responses (by IL-6) may explain the higher performance achieved by SAPS 3 + IL-6 vs. SAPS 3. Thus, implementation of IL-6 in critical care scores can improve prediction outcomes, especially in patients experiencing acute inflammatory conditions; however, statistical results may vary across hospital types and/or patient populations with different case mix.

## Introduction

Prediction of survival in critically ill patients is of crucial importance in modern critical care medicine [1]. Since crude mortality rates consider neither preexisting co-morbidities nor disease severity, they are not feasible for comparing outcomes across different intensive care units (ICU) or treatments. Therefore, mortality risk prediction scores have been developed, which assess survival probability based on grades of disease severity and other specific prerequisites or predisposing conditions in critically ill patients upon admission to an ICU. To date, the Simplified Acute Physiology Score (SAPS) 2 and SAPS 3 are two of the most extensively validated scores in critical care patients worldwide [2–6]. Such scores generate predicted mortality rates, which take into account different covariates by indirect standardization. This, in turn, allows calculation of standardized mortality ratios (SMRs) by dividing observed mortality rates to predicted mortality rates. While it is not the intention to predict the individual mortality risk of a patient, Intensive care scores can assess comprehensively the disease severity of a whole population. Provided an high accuracy of such scores, they can provide valuable input in decision-making processes, such as evaluation of new therapies, interventions or clinical management in intensive care settings [7–9].

Both scores, the SAPS 2 and SAPS 3, comprise various systemic organ dysfunction-related parameters, i.e., serum urea or creatinine, urinary output, serum bilirubin, oxygen partial pressure (Pa0_2_)/fraction of inspired oxygen (Fi0_2_) ratio and Glasgow Coma Scale [10, 11]. Immunological activity is primarily reflected in both scores via measurements of body temperature, white blood cell count (WBC) and heart rate (S1 Table). However, according to previous research, these variables, which reflect physiological reactions to inflammatory stimuli, cannot detect life-threatening aberrant or dysregulated systemic host responses, which are causally linked to multiple organ dysfunctions and other adverse outcomes [12]. Therefore, it is assumable that the predictive value of scores considering organ dysfunction, may gain power by co-consideration of inflammatory and/or immunological markers. Thus, in this study, we aimed to evaluate whether Interleukin-6 (IL-6), Procalcitonin (PCT) or C-reactive protein (CRP) can improve performance of the SAPS 2 and SAPS 3 in predicting survival. To our best knowledge, this is the first study that investigated potential performance enhancement of the SAPS 2 and SAPS 3 via implementation of IL-6, PCT and CRP in a mixed population of critically ill medical patients.

CRP plasma concentrations increase over 4 to 12 hours and peak within 24 to 72 hours following an inflammatory stimulus, whereby the induced levels can exceed the baseline levels by a factor of more than a thousand times; in addition, CRP plasma levels may remain elevated for almost 2 weeks [13–15]. Although elevations of CRP plasma levels may be equally triggered by both, infectious as well as non-infectious conditions, CRP is routinely monitored in hospitalized patients receiving anti-infective treatments [16, 17].

Elevated PCT levels can be detected within 3 to 6 hours following various infectious [18] and non-infectious [19] challenges. Particularly, PCT serum levels over 100 pg/nl can be reached in cases of severe bacterial, fungal, or parasitic infection. However, high serum levels of PCT are also detectable after trauma and/or following major surgery; overall, viral infection or inflammation of a noninfectious origin have been associated with lower PCT serum levels on average [20, 21].

Systemic IL-6 levels already increase within one hour after induction of inflammatory responses, that is early before CRP-levels or body temperature changes get detectable [17, 22]. Compared to other cytokines, IL-6, which remains remarkably longer detectable in the blood (both serum and plasma), has become eligible for routine diagnostics as it can be easily (and cost-efficiently) quantified with the multiplex assay method [23]. Former studies showed that early IL-6 measurements reliably correlate with multiorgan failure rates, complicated clinical courses and mortality in association with critical care conditions, such as polytrauma [24, 25], abdominal-aortic surgery [26, 27], pancreatitis [28], sepsis [29–31], acute respiratory distress syndrome (ARDS) [32, 33], cardiogenic shock [34, 35] or neurologic disorders [36].

## Materials and methods

### Ethics statement

This non-interventional study protocol was approved by the local institutional review board of the University Hospital Essen (IRB: Ethik-Kommission am Universitätsklinikum Essen). Because of the observational design of this cohort study, the institutional review board waived the requirement for patients' informed consent.

### Design, setting, patients

The study was conducted at an interdisciplinary 22-bed ICU at the University Hospital Essen, Germany, an academic clinical institution with a nearly 1300-bed capacity. Ten ICU beds were managed by the clinic of neurology, twelve ICU beds were covered by five specialized departments for internal medicine (cardiology, gastroenterology/hepatology, nephrology, hematology, endocrinology, angiology). According to the proposed classification of the World Federation of Societies of Intensive and Critical Care Medicine (WFSICCM) [37], this ICU meets all level-3-criteria except for a formal ICU follow-up program and a nurse-to-patient ratio (NPR) of 1:1 or 1:2; the NPR of this ICU was 1:3. The ICU medical team involved six physicians (1 or 2 specialists, 4 or 5 attending hospitalists) who worked in 8- to 12-hour shifts as critical care physicians with 24-hour in house coverage.

Within an 18-month period, we recorded each patients´ characteristics, medical history, reasons for admission as well as the worst clinical conditions and laboratory values within the first hour after ICU-admission for the SAPS 3 [11] or within the first 24 hours after ICU-admission for the SAPS 2 [10]. The SAPS 2 includes 15 variables, i.e., 12 physiology variables, age, type of admission, and one variable related to underlying disease [10]. The SAPS 3 utilizes 20 variables, i.e., 5 variables regarding patient characteristics prior to admission, 5 variables regarding the circumstances of the admission, and 10 physiology variables [11]. Under “supporting information”, the authors provide a side-by-side overview of both scoring systems along with a detailed description of parameters included in each score (S1 Table). For SAPS 2, we calculated the predicted mortality using the according general equation [10], while for SAPS 3, we chose the North European Logit out of the available customized formulas for calculation [11].

During the study period from June 2006 until January 2008, 603 patients were admitted to the ICU. As already stated in a previous publication using the same study population [6], we excluded patients with one of the following criteria: younger than 18 years (n = 1), arteriovenous coronary bypass surgery within 2 weeks before admission (n = 9), less than 24 hours stay at the ICU (n = 22), readmission in the study period (n = 23). Besides the aforementioned criteria, we also had to exclude further 34 patients due to missing biomarkers IL-6, PCT and CRP. Finally, a sample size of 514 patients remained and was subjected to further statistical analysis. In-hospital mortality was the endpoint of this study. Furthermore, we revised the definition of the acute conditions acute kidney injury and respiratory failure. While the previous publication [6] only registered these conditions if they were originally leading to the ICU-admission, we retrospectively applied the KDIGO-criteria [38] to define an acute kidney injury and the Berlin definition to define respiratory failure [39].

### Measurement of IL-6, PCT, CRP

Throughout the study period (2006–2008), IL-6, PCT and CRP plasma levels were routinely measured in blood samples that were collected within the first hours after ICU-admission. After collection at room temperature, samples were directly transferred to the laboratory of the hospital, where they were processed on a 24-hour basis. IL-6 serum concentrations were measured using a solid phase, enzyme-labelled chemiluminescent immunometric assay (IMMULITE® 2000 XP*i*; Siemens healthcare GmbH, Erlangen, Germany). For PCT measurement, a 2-site sandwich immunoassay with direct chemiluminescent technology was used (ADVIA Centaur® XPT Immunoassay-System, Siemens healthcare GmbH, Erlangen, Germany). CRP concentrations were measured in a serum sample, using a turbidimetric immunoassay test (ADVIA® 1800 Clinical Chemistry System, Siemens Healthcare Diagnostic, Erlangen, Germany). The reference ranges for the biomarkers were as follows: IL-6<15 pg/ml, PCT 0–0.5 ng/ml and CRP<0.5 mg/dl.

### Statistical analysis

SPSS (version 21.0; SPSS Inc, Chicago, IL) and SAS software (version 9.4; SAS Institute Inc., Cary, NC) were used to perform statistical analysis. For descriptive statistics, absolute and relative frequencies were calculated for categorical parameters, whereas continuous parameters were characterized using the median (MD) as well as the first and third quartile (Q1, Q3). Inferential statistics to compare deceased with non-deceased patients included Fisher's Exact Test for categorical variables and the Mann-Whitney U test for continuous variables. Results were considered statistically significant when *p*≤0.05.

We calculated AUROCs and their respective 95% CIs, to describe the discrimination of the SAPS 2 and SAPS 3 and their corresponding extended versions when combined with IL-6, PCT or CRP [40]. The extension of the SAPS scores with the biomarkers was conducted on the base of a binomial logistic regression model for in-hospital mortality using the respective SAPS and biomarker data as explanatory variables. In a further step, the ROC analysis was performed considering the predicted probabilities obtained from this model. In order to compare the discrimination performance of either the SAPS 2 or SAPS 3 against their extended versions, the difference between their AUROCs was calculated; then, the DeLong test was applied and considered statistically significant if *p*≤0.05.

## Results

### Patients characterisitcs

In our cohort of 514 patients, the median age was 63 years, the majority were male (n = 317; 61.7%) and the median BMI was 26 kg/m². The median length of stay in the ICU was 2 days (Table 1), whereas the mean length of stay was 6.7 ± 13.2 days. The vast majority of patients were admitted via the department of cardiology (n = 294; 57.2%). Thus, numerous cases involved critical conditions at ICU admission such as acute coronary syndrome (n = 112; 21.8%), acute aortic syndrome (n = 33; 6.4%), monitoring after coronary intervention (n = 29; 5.6%), decompensated heart failure (n = 29; 5.6%), cardiac arrhythmias (n = 21; 4.1%) and pulmonary hypertension (n = 15; 2.9%). After application of the KDIGO-criteria for acute kidney injuries [38] and the Berlin definition for respiratory failures [39], both diagnoses accounted for the most frequent acute conditions amongst all patients (acute kidney injury: n = 209; 40.7%, acute respiratory failure: n = 193; 37.5%) (Table 1). Further acute conditions, i.e., gastrointestinal bleeding (n = 30; 5.8%), sepsis (according to Sepsis-2 criteria [41], n = 28; 5.4%) or liver failure (n = 25; 4.8%), were less represented in the present study. One third of all patients underwent invasive mechanical ventilation (n = 171; 33.3%), while hemodialysis was applied in less than 15% of all cases (n = 76; 14.8%) (Table 1).

**Table 1:** Patient characteristics at admission overall and by survival status.

Parameters	All Patients	Survivals	Deaths	*P*
	MD [Q1;Q3] or n, (range: min-max) or (% of all patients)	MD [Q1;Q3] or n, (range: min—max) or (% of Survivals)	MD [Q1;Q3], (range: min—max) or (% of Deaths)	Significance
**Patients**	514	394	120	NA
**Age (y)**	63 [49;73] (range: 18–93)	64 [49;73], (range: 18–91)	61 [49;70], (range:19–93)	0.7051*
**Sex, n (%)**	♂ 317 (61.7%)	♂248 (62.9%)	♂ 69 (57.5%)	0.2858°
	♀ 197 (38.3%)	♀146 (37.1%)	♀ 51 (42.5%)	
**Body Mass Index (kg/m²)**	26 [23;28], (range: 16–47)	26 [23;29], (range: 16–47)	25 [23;28], (range: 16–43)	0.1654*
**Stay in the ICU (d)**	2 [1;7], (range: 0–153)	2 [1;5], (range: 0–81)	5 [2;12], (range: 0–153)	<0.0001*
**Acute conditions**				
**Acute kidney injury**	209 (40.7%)	129 (32.7%)	80 (66.7%)	<0.0001°
**Acute respiratory failure**	193 (37.5%)	112 (28.4%)	81 (67.5%)	<0.0001°
**Acute coronary syndrome**	112 (21.8%)	100 (25.4%)	12 (10%)	0.0002°
**Monitoring after surgery**	52 (10.1%)	47 (11.9%)	5 (4.2%)	0.0144°
**Acute aortic syndrome**	33 (6.4%)	25 (6.3%)	8 (6.7%)	0.8347°
**Gastrointestinal bleeding**	30 (5.8%)	18 (4.6%)	12 (10.0%)	0.0422°
**Monitoring after coronary intervention**	29 (5.6%)	28 (7.1%)	1 (0.8%)	0.0058°
**Decompensated heart failure**	29 (5.6%)	23 (5.8%)	6 (5.0%)	0.8249°
**Sepsis**	28 (5.4%)	12 (3.0%)	16 (13.3%)	0.0001°
**Liver failure**	25 (4.8%)	9 (2.3%)	16 (13.3%)	<0.0001°
**Cardiac arrhythmias**	21 (4.1%)	19 (4.8%)	2 (1.7%)	0.1862°
**Pulmonary arterial hypertension**	15 (2.9%)	14 (3.5%)	1 (0.8%)	0.2109°
**Acute pulmonary embolism**	10 (1.9%)	9 (2.3%)	1 (0.8%)	0.4656°
**Acute abdomen**	8 (1.6%)	4 (1.0%)	4 (3.3%)	0.0905°
**Intoxication**	5 (0.9%)	4 (1.0%)	1 (0.8%)	1.0000°
**Others**	62 (12.1%)	47 (11.9%)	15 (12.5%)	0.8733°
**Therapeutic interventions at admission**				
**Hemodialysis**	76 (14.8%)	38 (9.6%)	38 (31.7%)	<0.0001°
**Invasive mechanical ventilation**	171 (33.3%)	79 (20.0%)	92 (76.7%)	<0.0001°
**Scores and biomarkers at admission**				
**SAPS 2**	33 [22;47], (range: 5–118)	29 [19;39], (range: 5–93)	52 [41;66], (range: 13–118)	<0.0001*
**SAPS 2 predicted mortality**	14.0% [4.6;40.8], (range: 0.3–99.6)	9.6% [3.3;22.5], (range: 0.3–97.3)	50.7% [26.6;78.4], (range: 1.5–99.6)	<0.0001*
**SAPS 3**	58 [46;70], (range: 0–127)	54 [44;65], (range: 0–114)	72 [60;86], (range: 31–127)	<0.0001*
**SAPS 3 predicted mortality**	31% [12.5;57.0], (range: 0.0–98.0)	24.0% [10.0;46.0], (range: 0.0–96.0)	60.0% [35.5;81.0], (range: 2.0–98.0)	<0.0001*
**IL-6 (pg/ml)**	43.5 (13.7;152.7), [range: 0–91854]	26.6 (11.1;78.9), [range:0–50774]	245.5 (61.2;1812), [range:0.1–91854]	<0.0001*
**PCT (ng/ml)**	0.3 (0.1;1.9), [range: 0.01–498]	0.1 (0.6;8.6), [range:0.01–300.7]	2.2 (0.5;8.7), [range:0.03–498]	<0.0001*
**CRP (mg/dl)**	3.1 (0.8;10.2), [range: 0.01–52.7]	2.3 (0.6;8.6), [range:0.01–52.7]	6.6 (2.8;16.3), [range: 0.1–52]	<0.0001*

MD = median, Q1 = first quartile, Q3 = third quartile, n = count, NA = not applicable,

* = Mann-Whitney U test,

° = Fisher's exact test.

### Performance of the SAPS 2, SAPS 3 and biomarkers IL-6, PCT, CRP

The observed in-hospital mortality was 23.3% (n = 120 patients). The median score (MD [Q1; Q3]) was 33 [22; 47] for the SAPS 2 and 58 [46; 70] for the SAPS 3, resulting in median predicted mortalities of 14.0% [4.6%; 40.8%] and 31.0% [12.5%; 57.0%], respectively (Table 1). Corresponding mean values and standard deviations were 36.5 ± 19.5 for SAPS 2 and 59.4 ± 18.4 for SAPS 3, resulting in mean predicted mortalities of 25.7% ± 27.7 and 36.5% ± 27.3, respectively (S2 Table).

Both scores, i.e., the SAPS 2 and SAPS 3, exhibited an acceptable discrimination performance. However, the SAPS 2 showed a superior discrimination performance trend compared to SAPS 3 with an area under the receiver operating characteristic curve (AUROC) of 0.81 (95% confidence interval (CI), 0.76–0.86) compared to an AUROC of 0.72 (95% CI, 0.66–0.78), respectively (Table 2).

**Table 2:** Area under the receiver operating characteristic curve (AUROC) with 95% confidence interval (CI) for the SAPS 2 + SAPS 3, the biomarkers IL-6, PCT + CRP, and the extended SAPS 2 + SAPS 3 versions after combination with biomarkers.

	AUROC (95% CI)
**SAPS 2**	0.81 (0.76–0.86)
**SAPS 3**	0.72 (0.66–0.78)
**IL-6 (pg/ml)**	0.75 (0.69–0.81)
**PCT (ng/ml)**	0.72 (0.66–0.77)
**CRP (mg/dl)**	0.65 (0.59–0.72)
**SAPS 2 + IL-6**	0.82 (0.77–0.87)
**SAPS 2 +PCT**	0.81 (0.76–0.86)
**SAPS 2 +CRP**	0.81 (0.76–0.86)
**SAPS 3 + IL-6**	0.76 (0.69–0.81)
**SAPS 3 + PCT**	0.73 (0.67–0.78)
**SAPS 3 + CRP**	0.74 (0.68–0.80)

AUROC = area under the receiver operating characteristic curves; CI = 95% Wald Confidence Limits.

**Fig 1:**
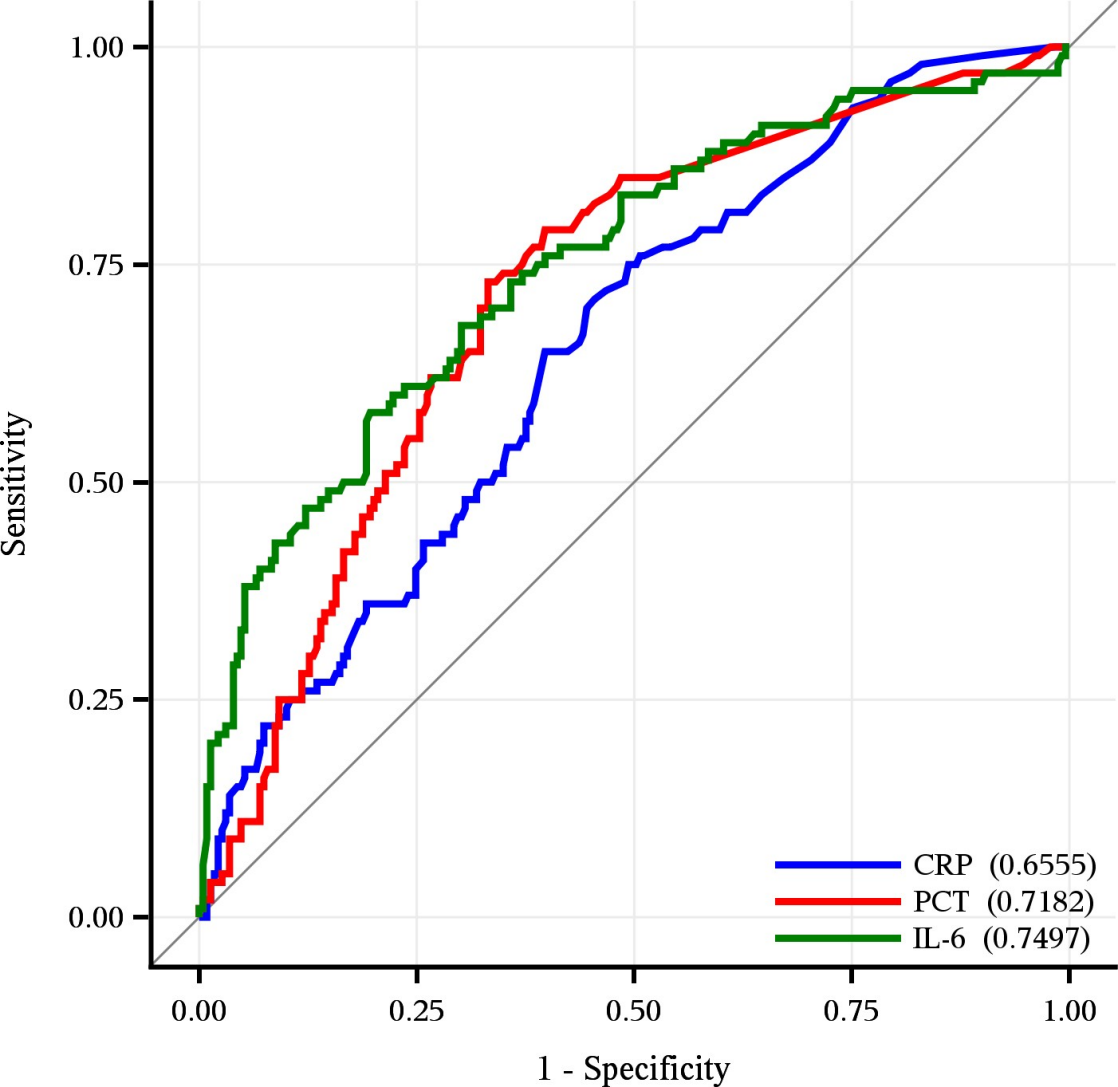
Discrimination performance of IL-6, PCT and CRP. All biomarkers showed an acceptable discrimination, however IL-6 with an AUROC of 0.75 (95% CI, 0.69–081) and PCT with an AUROC of 0.72 (95% CI, 0.66–077) demonstrated a significantly better discrimination power compared to CRP with an AUROC of 0.65 (95% CI, 0.59–072).

The median plasma concentration (MD [Q1; Q3]) of IL-6 was 43.5 pg/ml [13.7; 152.7], of PCT 0.3 ng/ml [0.1; 1.9] and of CRP 3.1 mg/dl [0.8; 10.2] (Table 1). These biomarkers also achieved an acceptable discrimination performance with AUROCs of 0.75 (95% CI, 0.66–0.78) for IL-6, 0.72 (95% CI, 0.66–0.77) for PCT and 0.65 (95% CI, 0.59–0.72) for CRP. However, the discrimination performance of CRP was significantly inferior when compared to IL-6 or PCT (Table 3 and Fig 1). None of these biomarkers performed better than the SAPS 2, while only IL-6 exceeded the discrimination power of the SAPS 3.

**Table 3:** Difference in the area under the receiver operating characteristic curve between the biomarkers, and between the original scores and their biomarker-extended versions.

Contrast	Estimate (CI)	P
**IL-6 –CRP**	0.0943 (0.0314–0.1572)	0.0033
**PCT–CRP**	0.0628 (0.00226–0.1232)	0.0420
**SAPS2+IL-6—SAPS2**	0.00932 (-0.00192–0.0206)	0.1042
**SAPS2+PCT—SAPS2**	0.00279 (-0.00282–0.00841)	0.3294
**SAPS2+CRP—SAPS2**	-0.00199 (-0.0148–0.0109)	0.7617
**SAPS3+IL-6—SAPS3**	0.0320 (0.00973–0.0543)	0.0049
**SAPS3+PCT—SAPS3**	0.00476 (0.000227–0.00929)	0.0396
**SAPS3+CRP—SAPS3**	0.0207 (-0.00280–0.0433)	0.0841

CI = 95% Wald Confidence Limits, p = DeLong test.

**Fig 2:**
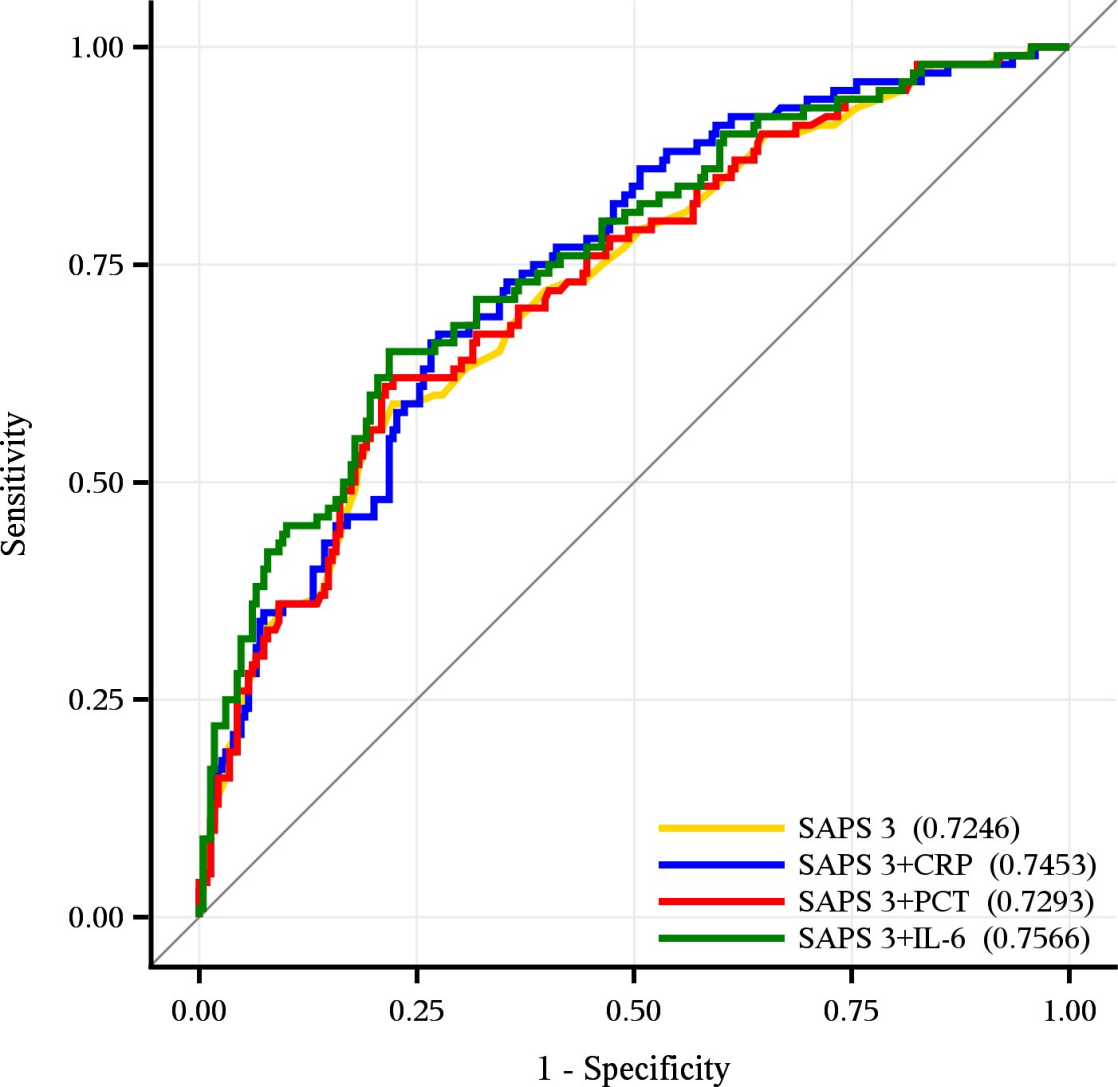
Discrimination performance of the SAPS 3 and the combined models SAPS 3+CRP, SAPS3+PCT and SPAS 3+IL-6. The SAPS 3 showed an AUROC of 0.72 (95% CI, 0.66–0.78). The SAPS 3 combined with IL-6 or PCT revealed significant improvements with AUROCs of 0.76 (95% CI, 0.69–0.81) and 0.73 (95% CI, 0.67–0.78) respectively. The combined SAPS 3+CRP model led to an AUROC of 0.74 (0.68–0.80).

**Fig 3:**
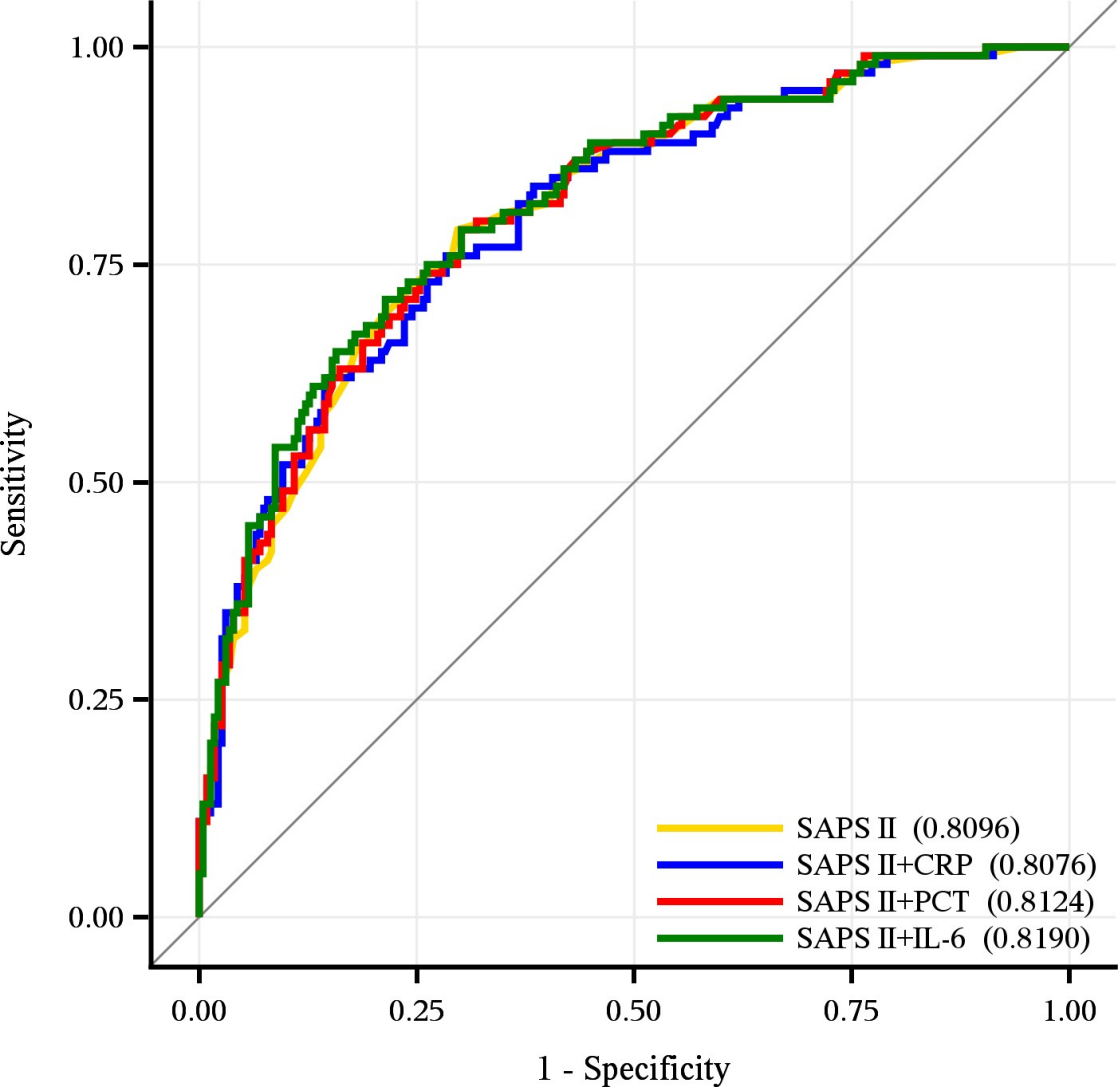
Discrimination performance of the SAPS 2 and the combined models SAPS 2+CRP, SAPS 2+PCT and SPAS 2+IL-6. The SAPS 2 showed an AUROC of 0.81 (95% CI, 0.76–0.86). The SAPS 2+IL-6 model showed an AUROC of 0.82 (95% CI, 0.77–0.87), whereas the SAPS 2+PCT and SAPS 2+CRP models displayed AUROCs of 0.81 (0.76–0.86).

When combined with the aforementioned biomarkers, improvements in discrimination performance were observed for both, the SAPS 2 and 3 (Table 3). However, statistically significant improvements of the AUROCs were only achieved when PCT or IL-6 were added to the SAPS 3, whereby these “hybrid” versions of the SAPS 3 still performed worse than the original SAPS 2 (Tables 2 and 3 and Fig 2). In detail, the combination of the SAPS 2 with IL-6 delivered the best discrimination performance with an AUROC of 0.82 (95% CI, 0.77–0.87), followed by an almost equal performance of SAPS 2 on its own or in combination with PCT and CRP, respectively (Tables 2 and 3 and Fig 3).

## Discussion

A recent research by Keegan et al. including more than 2500 critical care patients suggested that scoring systems with more predictor variables likely achieve better overall performances relative to those with fewer variables [42]. These results are in contrast to the higher accuracy in mortality prediction that was achieved by the SAPS 2 compared to the SAPS 3 in the present study. Even though SAPS 3 employs more variables than SAPS 2, the SAPS 2-related results of the present work are still in line with the findings of a prospective observational study with 3661 patients from more than 100 Italian intensive care units [4]. Nevertheless, alike the postulations by Keegan et al., we observed an improved discrimination performance of the SAPS 3 when combined by the additional variables IL-6, PCT or CRP and also of the SAPS 2, albeit only in combination with IL-6 (Table 2).

To the authors´ best knowledge, this study represents the first scientific effort to compare the discrimination performance between IL-6, PCT and CRP with regard to mortality prediction in a mixed population of critically ill medical patients. To date, the use of PCT and CRP in predicting sepsis or sepsis-related mortality or other adverse outcomes has been evaluated exclusively in infectious or septic patients [29, 43–48]. However, the SAPS scores are supposed to be applicated in any critical care setting.

In contrast to PCT and CRP, the predictive capacity of IL-6 has been studied in diverse intensive care contexts, showing overall good performances regarding prediction of mortality or other adverse outcomes [24–36]. Thus, IL-6 seems to be a rather reliable marker of illness severity and mortality in association with acute inflammatory responses [49]. The present data attest to the superior accuracy of IL-6 serum or plasma levels in predicting critical illness-related mortality. In fact, IL-6—as a marker—exceeded the predicitve perfomance of CRP or PCT (Table 2 and Fig 1) and also the discrimination performance of SAPS 3 (Table 2) in our cohort of predominantly non-septic patients.

However, these results need further external validation, since application of single parameters must be considered susceptible to errors. For example, IL-6 is prone to be influenced by specific patient characteristics. In more detail, gender, obesity, alcohol abuse and recent exercise or training seem to influence IL-6 serum levels [50–53]. Moreover, the impact of comorbidities or medications on IL-6-levels has not yet been sufficiently examined.

As proper processing of blood samples is a crucial pre-analytical step for the integrity of biomarker-related results, the techniques of specimen collection and laboratory processing in the present study deserves further discussion. First, blood samples were transferred uncooled; therefore, they were processed in less than 6 hours from blood draw [54]. Otherwise, laboratory tests would become susceptible for biased measurements due to ongoing IL-6 production through activated leukocytes [55] or proteolytic degradation and temperature lability [56]. Second, the herein considered biomarkers were only measured during the routinely obtained blood samples at ICU-admission. This procedure fits the requirements of the SAPS 3 in terms of parameters to be collected only within the first hour before or after ICU admission. However, SAPS 2 requires the worst parameters within the first 24 hours after initiation of ICU treatment. As half-life period and peak time of the biomarkers strongly vary, a more frequent monitoring of biomarkers throughout the first 24 hours could differently affect the discriminative performance of these biomarkers. This limitation should be addressed in future research.

Finally, characteristics that may have influenced score performances in our single-center study are the predominant fraction of patients with critical cardiovascular events, the low proportion of septic patients and the rather unfavorable nurse-to-patient ratio of 3:1. Since the validation of intensive care scores always dependent on specific case mixes, different admission and discharge criteria, diversity in hospital care and heterogeneous staff- or shift-work patterns, we hope that our research encourages clinicians to re-evaluate and further validate the herein presented findings in other cohorts and intensive care settings.

## Conclusions

In the present study, the SAPS 2 exerted a superior discrimination performance relative to the SAPS 3. Among the analyzed markers, IL-6 achieved the highest discrimination performance over PCT and CRP. Discrimination performance of SAPS 3 improved significantly when combined with IL-6 and PCT. The combined SAPS 2/IL-6 model showed the best overall discrimination performance, albeit not significantly different in comparison to the SAPS 2.

Previous studies suggested that IL-6 could reliably indicate illness severity during the course of acute inflammatory responses [24–36]. Therefore, it seems likely that systemic IL-6 concentrations can assess immunological activity and thus reflect disease severity in critical care patients. To date, IL-6 has not been incorporated yet in any of the broadly validated predictive scoring systems for critically ill patients such as Apache II and III [57, 58], SAPS 2 [10], SAPS 3 [11], MPM-II &-III [59, 60] or SOFA [61].

Taken together, our data stress out the beneficial effect of IL-6 on the SAPS 2 and SAPS 3 predictive performance. The low proportion of septic patients at admission in our non-selected cohort of medical critical care patients even indicate the broad range of applicability of supplementing IL-6 to risk stratification tools in intensive care medicine. Even though immunological processes during critical illness are not completely elucidated yet, it is assumable that systemic inflammatory response syndromes (SIRS) and counter regulatory response syndromes (CARS), which may facilitate early organ failure, are not sufficiently assessed by body temperature, white blood cell count and heart rate or other parameters employed by scores such as the SAPS2 or the SAPS3. Since intensive care scores like the SAPS 2 or SAPS 3 are prone to be outdated due to changes in ICU-populations, evolution of diagnostic, therapeutic, technological and economic aspects, the implementation of IL-6 in intensive care scores may be a valuable contribution towards a modern and precise risk stratification method among heterogeneous critical care patients and settings.

## Supporting information

S1 Table Simplified Acute Physiology Score (SAPS) 2 and SAPS 3. (DOCX)

S2 Table Case specifications at ICU-admission with corresponding SAPS 2& SAPS 3 mean scores and SMRs as well as corresponding biomarkers IL-6, PCT & CRP median values. (DOCX)
