# Supplementary material for: Score performance of SAPS 2 and SAPS 3 in combination with biomarkers IL-6, PCT or CRP
Source: PLoS One. 2020 Sep 3;15(9):e0238587. doi: 10.1371/journal.pone.0238587 (PMC7470390; doi:10.1371/journal.pone.0238587)
Supplement: S1 Table — (DOCX) [file pone.0238587.s001.docx]

**S1 Table**. Simplified Acute Physiology Score (SAPS) 2 and SAPS 3

| **SAPS 2**:  worst values for clinical and laboratory parameters within the past 24h after ICU-admission | | | | **SAPS 3**:  worst values for clinical and laboratory parameters within the first hour after ICU-admission | | | | | | | | | |
| --- | --- | --- | --- | --- | --- | --- | --- | --- | --- | --- | --- | --- | --- |
| **Clinical parameters** | | | | | | | | | | | | | |
| 1. | Age [years]: | <40: 0p; 40-59: 7p; 60-69: 12p; 70-74: 15p; 75-79: 16p; ≥80: 18p | | 1. | | Age [years] | | | | | <40: 0p; ≥40-60: 5p; ≥60-<70: 9p; ≥70-<75: 13p; ≥75-<80: 15p; ≥80: 18p | | |
| 2. | Heart rate [bpm]: | <40: 11p; 40-69: 2p; 70-119: 0p; 120-159: 4p; ≥160: 7p; | | 2. | | Heart rate, [bpm] | | | | | <120: 0p; ≥120-<160: 5p; ≥160: 7p | | |
| 3. | SBP [mmHg]: | <70: 13p; 70-99: 5p; 100-199: 0p; ≥200: 2p | | 3. | | SBP [mmHg] | | | | | <40: 11p; ≥40-<70: 8p; ≥70-<120: 3p; ≥120: 0p | | |
| 4. | Body Temperature [°C]: | <39: 0p; ≥39: 3p | | 4. | | Body Temperature [°C] | | | | | <35: 7p; ≥35: 0p | | |
| 5. | Glasgow Coma Scale*: | <6: 26p; 6-8: 13p; 9-10: 7p; 11-13: 5p; 14-15: 0p | | 5. | | Glasgow Coma Scale* | | | | | 3-4: 15p; 5: 10p; 6: 7p; 7-12: 2p; ≥13: 0p | | |
| 6. | Urine output [mL/24h]: | <0.5: 11p; 0.5-0.99: 4p; ≥1: 0p | |  |  |  |  |  |  |  |  |  |  |
| **Respiratory parameters** | | | | | | | | | | | | | |
| 7. | If Mechanical Ventilation/CPAP or Pulmonary Artery Catheter in past 24h: PaO2/FiO2<100: 11p; PaO2/FiO2 100-199: 9 p; PaO2/FiO2≥200: 6 p | | | 6. | | If Mechanical Ventilation: paO2/FiO2<100: 11p; PaO2/FiO2 ≥100: 7p;  No Mechanical Ventilation: PaO2 <60: 5p; PaO2 ≥60: 0p | | | | | | | |
| **Laboratory parameters** | | | | | | | | | | | | | |
| 8. | Bicarbonate [mmol/L]: | | <15: 6p; 15-19: 3p; ≥20: 0p | 7. | | Creatinine, [mg/dL] | | | | | <1.2: 0p; ≥1.2-<2: 2p; ≥2-<3.5: 7p, ≥3.5: 8p | | |
| 9. | BUN [mg/dL]: | | <60: 0p; 60-179: 6p; ≥180: 10p | 8. | | pH: | | | | | ≤7.25: 3p; >7.25: 0p | | |
| 10. | Total Bilirubine [mg/dL]: | | <4.0: 0p; 4.0-5.9: 4p; ≥6.0: 9p | 9. | | Total Bilirubine [mg/dL] | | | | | <2: 0p; ≥2-<6: 4p; ≥6: 5p | | |
| 11. | WBC [x10^9^/L]: | | <1.0: 12p; 1.0-19.9: 0p; ≥20: 3p | 10. | | WBC [x10^9^/L] | | | | | <15: 0p; ≥15: 2p | | |
| 12. | Potassium [mmol/L]: | | <3: 3p; 3.0-3.9: 0p; ≥5: 3p | 11. | | Plateletes [G/L] | | | | | <20: 13p; ≥20-<50: 8p; ≥50-<100: 5p; ≥100: 0p | | |
| 13. | Sodium [mmol/L]: | | <125: 5p; 125-144: 0p; ≥145: 1p |  |  |  |  |  |  |  |  |  |  |
| **Chronic diseases/Co-Morbidities** | | | | | | | | | | | | | |
| 14. | Metastatic Cancer: 9p; Hematologic malignancy: 10p; AIDS: 17p | | | 12. | | Cancer Therapy(e.g. Chemotherapy, Immunosuppression other, Radiotherapy, Steroid treatment): 3p; Metastatic Cancer: 11p; Haematological Cancer: 6p; Chronic Heart Failure (NYHA IV): 6p; Cirrhosis: 8p, AIDS: 8p | | | | | | | |
| **Admission-related Parameters** | | | | | | | | | | | | | |
| 15.  . | Type of admission: | | Medical: 6p; Scheduled surgical: 0p, Unscheduled surgical: 8p | 13. | | ICU-admission | | | | Planned: 16p; Unplanned: 19p | | | |
|  |  |  |  | 14. | | Surgical Status at ICU Admission | | | | No surgery: 5p; Scheduled surgery: 0p; emergency surgery: 6p | | | |
|  |  |  |  | 15. | | Anatomical Site of Surgery | | - Transplantation surgery (Liver, Kidney, Panreas, Kidney and Pancreas, others): -11p - Trauma (includes Thorax, Abdomen, limb or multiple): -8p ; - Cardiac surgery (CABG without valvular repair): -6p; - Neurosurgery ( Cerebrovascular accident): 5p; All others: 0p | | | | | |
|  |  |  |  | 16. | | Length of stay before ICU admussion [days] | | | | | | | <14: 0p; ≥14-<28: 6p; ≥28: 7p |
|  | | | | 17. | | Intra-hospital location before ICU admission | | | | | | | Emergency room: 5p; Other ICU: 7p; Other Ward: 8p |
|  |  |  |  | 18. | | Vasoactive drugs before ICU admission | | | | | | Yes: 3p; No: 0p | |
|  |  |  |  | 19. | | Reason(s) for ICU admission | - Cardivascular: Rhythm disturbances (without simultaneous occurence of seizures): -5p; Hypovolemic hemorrhagic shock: 3 points, Hypovolemic non-hemorrhagic shock: 3p; Septic shock: 5p; Anaphylactic shock, mixed and undefinded shock: 5p; All others: 0p - Hepatic: Liver failure: 6p; All others: 0p - Digestive: Severe pancreatitis: 9p; Acute abdomen, Other: 3p ; All others: 0p - Neurologic: Intracranial mass effect: 10p; Focal neurologic deficit: 7p; Seizures: -4p; Coma, Stupor, Obtuned patient, Vigilance disturbance, Confusion, Agitation Delirium: 4p; All others: 0p | | | | | | |
|  |  |  |  | 20. | Acute infection at ICU admission | | | | Nosocomial: 4p; Respitratory: 5p; All Others 0p | | | | |
| **Logit=-7,7631+(0,0737*SAPS2)+(0,9971*ln(SAPS2+1)); Probability of Mortality: e^logit^/(1+e^logit^)** | | | | **North European Logit: -26.9065+ln(SAPS3+5.5077)*6.; Probability of Mortality: e^logit^/(1+e^logit^)** | | | | | | | | | |

p=points; Glascow Coma Scale=GCS, *if patient is currently sedated, use estimated GCS prior to sedation; BUN=Blood Urea Nitrogen, WBC=White Blood Cell Count, SBP=Systolic Blood Pressure; bpm=beats per minute; pH=Hydrogen in concentration. Modified by Jahn et al. 2019 [3].
