# Supplementary material for: Score performance of SAPS 2 and SAPS 3 in combination with biomarkers IL-6, PCT or CRP
Source: PLoS One. 2020 Sep 3;15(9):e0238587. doi: 10.1371/journal.pone.0238587 (PMC7470390; doi:10.1371/journal.pone.0238587)
Supplement: S2 Table — (DOCX) [file pone.0238587.s002.docx]

**S2 Table.** Case specifications at ICU-admission with corresponding SAPS 2& SAPS 3 mean scores and SMRs as well as corresponding biomarkers IL-6, PCT & CRP median values.

| **Statistics** | **Total** | **Deaths** | **SAPS 2** | | **SAPS 3** | | **IL-6 (pg/ml)**  **MD [Q1;Q3]** | **PCT (ng/ml) MD [Q1;Q3]** | **CRP (mg/dl) MD [Q1;Q3]** |
| --- | --- | --- | --- | --- | --- | --- | --- | --- | --- |
|  |  |  | **Mean ± SD** | **SMR (95% CI)** | **Mean ± SD** | **SMR (95% CI)** |  |  |  |
| **All** | 514 | 120 (23.3%) | 36.5 ± 19.5 | 0.91 (0.83-0.99) | 59.4 ± 18.4 | 0.64 (0.59-0.68) | 43.5 (13.6;152.7) | 0.3 (0.1;1.9) | 3.1 (0.8;10.2) |
| **Cardiological Fraction** | 294 | 42 (14.3%) | 32.2± 16.3 | 0.73 (0.64-0.85) | 54.3 ± 15.7 | 0.49 (0.45-0.54) | 26.9(11.1;84.2) | 0.1(0.1;0.5) | 2.1(0.6;6.9) |
| **Gastroenterological Fraction** | 83 | 30 (36.1%) | 40.4 ± 21.2 | 1.16 (0.97-1.45) | 68.1 ± 19.7 | 0.71 (0.63-0.81) | 80.0(30.5;538.0) | 1.2(0.4;3.8) | 3.1(1.3;6.3) |
| **Nephrological Fraction** | 55 | 30 (54.4) | 50.2 ± 23.0 | 1.25 (1.05-1.55) | 75.4 ± 20.4 | 0.93 (0.83-1.06) | 209.0(40.5;2806.0) | 2.2(0.4;12.3) | 14.2(5.6;21.7) |
| **Hematooncological Fraction** | 36 | 8 (22.2%) | 41.6 ± 18.2 | 0.66 (0.52-0.93) | 58.4 ± 15.9 | 0.63 (0.51-0.82) | 75.5(20.3;132.5) | 1.6(0.6;4.1) | 6.5(1.8;16.1) |
| **Endocrinological/Other Fractions** | 46 | 10 (21.7%) | 37.3 ± 22.02 | 0.75 (0.57-1.08) | 57.0 ±16.1 | 0.64 (0.53-0.81) | 61.3(21.1;176.5) | 0.1(0.1.;1.3) | 4.7(0.6;13.6) |
| **Acute Kidney Injury** | 209 | 80 (38.3%) | 45.8 ± 19.9 | 1.01 (0.91-1.13) | 66.2 ± 18.9 | 0.81 (0.75-0.89) | 75.5 (21.2;249.0) | 1.1 (0.2;8.3) | 3.6 (1.4;13.3) |
| **Acute Respiratory Failure** | 193 | 81 (42.9%) | 51.5 ± 20.1 | 0.91 (0.84-1.01) | 69.3 ± 18.3 | 0.83 (0.77-0.89) | 96.0 (31.1;393.0) | 1.1 (0.1;5.8) | 6 (2.3;14.6) |
| **Acute coronary syndrome** | 112 | 12 (10.7%) | 30.2 ± 16.7 | 0.59 (0.47-0.79) | 53.2 ± 14.8 | 0.39 (0.34-0.46) | 20.8 (10.7;58.5) | 0.1 (0.1;0.3) | 1.6 (0.6;4.7) |
| **Monitoring After Surgery** | 52 | 5 (9.6%) | 34.8 ± 17.1 | 0.40 (031-0.56) | 55.9 ± 16.5 | 0.30 (0.24-0.38) | 75.9 (39.8;157.0) | 0.3 (0.1;1.3) | 4.2 (1.3;12.2) |
| **Acute Aortic Syndrome** | 33 | 8 (24.2%) | 32.7 ± 15.5 | 1.22 (0.88-1.97) | 50.6 ± 13.7 | 1.03 (0.80-1.43) | 53.7 (28.2;159.5) | 0.2 (0.1;1.4) | 5.9 (2.1;15.1) |
| **Gastrointestinal Bleeding** | 30 | 12 (40.0%) | 34.9 ± 13.5 | 1.77 (1.38-2.46) | 62.7 ± 16.9 | 0.93 (0.76-1.21) | 43.4 (12.1;90.3) | 0.5 (0.3;1.8) | 2.9 (0.9;4.8) |
| **Monitoring After Coronary Interv.** | 29 | 1 (4.4%) | 27.8 ± 14.4 | 0.31 (0.21-0.63) | 56.9 ± 16.9 | 0.14 (0.11-0.19) | 18.4 (7.0;50.7) | 0.1 (0.1;0.1) | 0.8 (0.2;2.3) |
| **Decompensated Heart Failure** | 29 | 6 (20.7%) | 39.6 ± 19.5 | 0.71 (0.51-1.13) | 59.3 ± 17.7 | 0.59 (0.47-0.82) | 27.5 (11.4;120.7) | 0.5 (0.1;1.4) | 3.0 (0.8;6.9) |
| **Sepsis** | 28 | 16 (57.1%) | 50.3 ± 23.7 | 1.29 (1.08-1.77) | 73.5 ± 24.7 | 1.08 (0.88-1.39) | 2250 (531;10000) | 7.5 (3.5;45.3) | 19.5 (14.4;27.9) |
| **Liver Failure** | 25 | 16 (64.0%) | 52.7 ± 25.4 | 1.31 (1.04-1.79) | 77.56 ± 18.1 | 0.98 (0.85-1.16) | 137.5 (25.0;578.5) | 3.3 (1.7;6.7) | 2.7 (1.4;5.7) |
| **Cardiac Arrhythmias** | 21 | 2 (9.5%) | 34.1 ± 17.6 | 0.39 (0.27-0.85) | 53.3 ± 17.6 | 0.31 (0.23-0.53) | 20.7 (5.2;57.4) | 0.1 (0.1;0.2) | 0.9 (0.2;3.5) |
| **Pulmonary Arterial Hypertension** | 15 | 1 (6.7%) | 27.2 ± 6.6 | 0.72 (0.54-1.05) | 50 ± 10.2 | 0.34 (0.24-0.60) | 16.3 (6.8;21.4) | 0.1 (0.1;0.1) | 0.9 (0.2;2) |
| **Acute Pulmonary Embolism** | 10 | 1 (10.0%) | 34.6 ± 28.5 | 0.38 (0.21-3.33) | 56 ± 17.1 | 0.29 (0.21-0.57) | 27.6 (13.5;36.9) | 0.1 (0.1;0.2) | 3.4 (0.7;9.2) |
| **Acute Abdomen** | 8 | 4 (50.0%) | 48.1 ± 27.3 | 1.17 (0.72-3.16) | 57.6 ± 19.3 | 1.46 (0.89-4.02) | 890 (193;1666) | 10.3 (1.1;19.6) | 21.4 (8.8;24.2) |
| **Intoxication** | 5 | 1 (20.0%) | 31.2 ± 37.6 | 0.85 (0.33-1.49) | 52.6 ± 30.36 | 0.76 (0.33-2.54) | 2.8 (2.8;2.8) | 0.1 (0.1;0.1) | 0.2 (0.2;0.3) |
| **Others** | 62 | 15 (25.0%) | 37.8 ± 20.7 | 0.90 (0.71-1.23) | 61.5 ± 19.1 | 0.64 (0.54-0.77) | 36.2 (11.9;99.6) | 0.4 (0.1;1.8) | 2.6 (0.9;8.2) |

SD=Standard Deviation; SMR=Standard Mortality Ratio (=observed mortality rate/mean expected mortality rate); CI=Confidence Interval; MD=median, Q1=first quartile, Q3=third quartile
